# Arsenic and Stillingia in Diseases of the Skin

**Published:** 1857-09

**Authors:** 


*Arsenic and Stillingia in Diseases of the Skin.*—In the Boston Medical and Surgical Journal, we find in a communication from Wm. M. Cornell, M. D., of Boston, Sillingia recommended in cutanious diseases, and in secondary and tertiary syphilis. In cutanious diseases he thinks is decidedly preferable, and less dangerous than arsenic. The article closes with the following quotation from Dr. Williams: If we can safely spare our patients the infliction of heoric remedies, from the effects of which they may be months in recovering, we confer a great benefit on them, and obtain another triumph for our profession.

